# CANNE: CLIP-Based ANNE Selection for Noisy-Label Learning

**DOI:** 10.3390/e28080849

**Published:** 2026-07-30

**Authors:** Ge Jin, Qian Zhang, Li Huang, Yongqi Yuan, Yulong Qiao, De Yu, Mengjie Ye, Yingwen Zhu

**Affiliations:** School of Information Technology, Jiangsu Open University, Nanjing 210036, China; jinge@jsou.edu.cn (G.J.);

**Keywords:** deep neural networks, noisy labels, sample selection, vision–language model

## Abstract

Learning with noisy labels (LNL) remains challenging, especially when the identification of clean samples relies heavily on the predictions of the model being trained. In such cases, early-stage selection errors may be reinforced during iterative optimization, leading to unreliable supervision. To alleviate this issue, a two-stage framework, termed CANNE, is proposed by combining Contrastive Language–Image Pre-training (CLIP)-based conservative offline cleaning with Adaptive Nearest Neighbors and eigenvector-based sample selection (ANNE)-based online refinement. Specifically, a high-confidence clean seed set is first constructed using two complementary probability sources derived from frozen CLIP representations and reliability criteria, including class-wise loss modeling and prediction consistency. This seed set is then used as a set of reliable anchors during the subsequent ANNE training process, where online feature- and neighborhood-based refinement further recovers and adjusts sample partitions. In this way, CANNE uses external vision–language priors to provide conservative and persistent guidance while preserving the adaptive recovery ability of online noisy-label learning. Experimental results on CIFAR-10, CIFAR-100, Animal-10N, and Mini-WebVision, together with additional evaluation under open-set noise, show that the proposed method achieves competitive performance across diverse noisy-label settings. In particular, CANNE achieves 96.6% and 96.3% best accuracies on CIFAR-10 under 80% and 90% symmetric noise, respectively, and 81.0% and 79.0% on CIFAR-100 under 20% and 50% symmetric noise. Additional repeated-run, threshold-sensitivity, and runtime analyses further indicate that the CLIP-based seed set provides stable guidance with only moderate computational overhead.

## 1. Introduction

The rapid advancement of deep neural networks (DNNs) in computer vision has been largely supported by the availability of large-scale annotated datasets. However, collecting high-quality labels at scale is often costly and time-consuming. In practice, many datasets are therefore collected from web queries or crowdsourcing platforms, where label noise is almost unavoidable. Training directly on such corrupted annotations often leads to noticeable performance degradation, as over-parameterized DNNs tend to gradually memorize noisy labels during optimization [1]. Consequently, learning with noisy labels (LNL) has become an important problem in building robust visual recognition systems.

Over the past few years, a variety of strategies have been developed to address label noise, among which sample selection has emerged as one of the most effective directions. The main idea is to identify a subset of likely clean samples during training, often based on the small-loss phenomenon that DNNs tend to fit clean patterns earlier than noisy ones [2]. Despite substantial progress, most existing selection methods still rely heavily on the predictions of the network being trained. This gives rise to a practical chicken-and-egg dilemma: robust classification requires reliable clean samples, while the identification of clean samples itself depends on the reliability of the classifier. During the early stages of training, the model may remain insufficiently discriminative, causing false sample selection. Once such errors are fed back into the optimization loop, confirmation bias can gradually accumulate and eventually impair performance, especially under severe or open-set noise conditions.

To mitigate this issue, additional information that is less coupled with the noisy labels of the target training set can be beneficial. Recently, vision–language models (VLMs), such as CLIP [3], have shown strong zero-shot recognition ability by learning transferable image–text representations from large-scale pre-training. Since CLIP is trained independently of the target noisy dataset, its semantic predictions are not directly produced by the in-training classifier and can therefore provide an external source of reliability estimation. This property has motivated recent studies, such as CLIPCleaner [4], to use CLIP for offline clean-sample selection in noisy-label learning. CLIPCleaner demonstrates that CLIP can provide a useful external reliability estimate for identifying likely clean samples. Nevertheless, CLIP-based cleaning alone is not always sufficient for the entire noisy-label learning process. Zero-shot predictions may be affected by domain gaps, fine-grained category ambiguity, or prompt sensitivity. Moreover, conservative CLIP-based selection usually prioritizes precision and may leave many clean but difficult samples unselected. These observations suggest that CLIP-based offline cleaning and online noisy-label refinement are complementary: the former provides reliable external seeds, while the latter can adapt to the target dataset and recover additional samples during training.

Motivated by this complementarity, a two-stage framework termed CANNE is proposed to connect conservative CLIP-based offline cleaning with ANNE-based online refinement. In the first stage, a high-confidence clean seed set is constructed using CLIP-derived reliability estimates. This seed set is intentionally conservative: it is expected to contain highly reliable samples, while some clean samples may remain unselected. In the second stage, these seeds are incorporated into ANNE [5], which dynamically refines sample partitions according to feature structure and neighborhood relationships. The CLIP-selected seeds act as reliable anchors during online refinement, helping reduce early-stage error accumulation while allowing the training model to adaptively recover additional samples from the remaining data.

The main contributions of this work are summarized as follows.

A two-stage noisy-label learning framework, termed CANNE, is proposed to integrate CLIP-based conservative offline clean-seed construction with ANNE-based online adaptive refinement. The offline stage combines two complementary reliability estimates derived from frozen CLIP representations, namely zero-shot image–text probabilities and visual-feature-induced probabilities, thereby reducing the exclusive dependence of early sample selection on the in-training classifier.

A persistent seed-guided refinement strategy is developed to preserve high-confidence CLIP-selected samples as reliable anchors during ANNE training, while samples outside the seed set remain subject to adaptive online selection. This design provides stable supervision during early refinement without removing the feature- and neighborhood-based sample recovery ability of ANNE.

Extensive experiments on CIFAR-10, CIFAR-100, Animal-10N, and Mini-WebVision, together with additional evaluation under open-set noise, show that the proposed method achieves competitive performance across diverse noisy-label settings, with particularly favorable results in several severe-noise scenarios.

## 2. Related Work

### 2.1. Learning with Noisy Labels

Learning with noisy labels has been widely studied because large-scale training data collected from web search engines, crowdsourcing platforms, or weakly supervised pipelines usually contain imperfect annotations. A central difficulty in this problem is that deep neural networks can gradually memorize corrupted labels during optimization [1]. As a result, standard training with cross-entropy loss often leads to degraded generalization when the noise ratio becomes high or the noise pattern becomes complicated.

Several types of methods have been developed to reduce the effect of noisy supervision. Robust loss functions attempt to make the training objective less sensitive to corrupted labels. Early studies analyzed learning under noisy labels from a risk correction perspective [6,7]. Generalized cross entropy was introduced to balance the optimization advantage of cross entropy and the robustness of mean absolute error [8], while symmetric cross entropy used a reverse cross-entropy term to improve noise tolerance [9]. Other objectives, such as normalized losses and active negative losses, were also investigated for more robust optimization under label noise [10,11]. Prediction-consistency regularization combined with contrastive clustering has also been explored to improve robustness to noisy supervision [12]. MixUp has been commonly used to regularize the decision boundary and reduce overfitting to individual noisy samples [13], and its relation to the vicinal risk minimization principle has also been studied theoretically [14]. These methods are usually simple and can be combined with other frameworks, but their standalone performance may be limited under severe noise.

Label correction is another important direction. Instead of directly discarding unreliable samples, noisy annotations are corrected or refined according to model predictions or estimated noise patterns. Joint optimization methods update labels and network parameters simultaneously [15], while probabilistic end-to-end correction methods refine noisy labels during training [16]. Progressive correction has also been studied for feature-dependent label noise [17]. More recent methods further combine label correction with contrastive or prototypical representations to improve the quality of corrected labels [18,19,20]. Other approaches, including reflective learning [21], part-level noisy-class posterior estimation [22], and uncertainty-guided label correction [23], provide additional mechanisms for refining noisy supervision. Although label correction can recover part of the corrupted supervision, its reliability is still closely tied to the quality of the model predictions. When the classifier is not sufficiently reliable, especially in early training, incorrect corrections may be introduced and reinforced.

Sample selection has therefore become one of the most influential strategies in LNL. The basic idea is to identify likely clean samples and use them as reliable supervision. Many sample-selection methods are based on the small-loss assumption, which indicates that clean samples are typically learned earlier and thus have lower losses than noisy samples at the beginning of training [24]. Co-teaching follows this principle by training two networks and allowing them to exchange selected small-loss samples [25]. DivideMix extends this strategy by fitting the sample-wise loss distribution with a Gaussian mixture model and treating samples with unreliable labels as unlabeled data in a semi-supervised learning framework [2]. Later studies improved sample selection through high-confidence sample mining [26], cyclical joint-loss and co-teaching strategies [27], dynamic instance-specific selection and correction [28], optimal transport-based filtering [29], uniform sample selection with contrastive learning [30], hyperspherical margin weighting [31], learning-risk-based separation of hard clean and noisy samples [32], and agreement-based co-regularization [33]. These methods have shown strong performance in many noisy-label settings.

However, most sample-selection methods still estimate sample reliability from the network being optimized, using its losses, predictions, or learned features. This dependency can be problematic in the early training stage, when the model is not yet discriminative. Once incorrectly selected samples are fed back into the training loop, the resulting confirmation bias may affect subsequent optimization. This concern is related to recent studies on prediction instability. Jauhari and Hidey [34] studied compute-efficient churn reduction for conversational agents by reducing undesirable output changes between model versions, while Datta et al. [35] measured and mitigated local instability in deep neural networks under input perturbations. Although their settings differ from LNL, these studies highlight that unstable model decisions can affect downstream decision-making. In noisy-label learning, such instability is particularly harmful because early incorrect clean/noisy partitions may be reinforced during training. This limitation motivates the use of additional information that is less coupled with the noisy labels of the target training set.

### 2.2. Feature-Based Sample Selection and Online Refinement

In addition to loss-based sample selection, feature-based selection has also been explored. Instead of relying only on classification losses or prediction confidence, these methods use the structure of the learned feature space to distinguish clean and noisy samples. The underlying assumption is that samples from the same semantic class tend to form compact or structured clusters, whereas mislabeled samples are more likely to deviate from the dominant class structure.

FINE detects clean samples by measuring the alignment between sample representations and class-wise dominant eigen-directions [36]. The dominant eigenvector of the feature distribution is used as a class-specific reference direction, and samples that are more consistent with this direction are more likely to be selected as clean. This provides a feature-space criterion beyond the loss value. Nevertheless, the quality of the selection still depends on whether the learned representation has become sufficiently meaningful.

Neighborhood-based methods further exploit local relationships in the feature space. SSR introduces a sample selection and relabeling framework based on nearest-neighbor voting and confidence-based relabeling [37]. In this framework, the local neighborhood structure provides evidence for whether a sample label is reliable. ANNE further follows a heterogeneous selection design by combining different strategies for samples with different confidence levels [5]. The training set is first partitioned into high-confidence and low-confidence subsets. Eigenvector-based selection is then applied to the high-confidence subset, while adaptive nearest-neighbor selection is used for the low-confidence subset. This heterogeneous design is useful because samples with different confidence levels may require different selection rules.

Several recent methods also adopt progressive or two-stage designs. LongReMix constructs a high-confidence clean set and uses it to guide subsequent robust training [26]. C2MT introduces a cross-to-merge training strategy with a class balance mechanism to improve robustness under noisy supervision [38]. PSSCL decouples clean-set discovery and robust training through progressive sample selection with contrastive learning [39]. These methods suggest that reliable initialization and adaptive refinement are both important for noisy-label learning.

TCC-net also adopts a two-stage design by combining contradictory loss, co-teaching, and meta-learning for training under noisy supervision [40]. More broadly, contrastive representation learning has provided an important foundation for robust feature learning. SimCLR [41] and MoCo [42] established representative self-supervised contrastive frameworks, while supervised contrastive learning extended this principle to label-aware representation learning [43]. These ideas were subsequently adapted to webly or noisily supervised learning through momentum prototypes [44], selective supervised contrastive learning [45], and semantic clustering with semi-supervised learning [46].

Despite these advances, the initial partitioning and refinement process in these methods is still mostly driven by the in-training model. When early predictions or representations are unreliable, the online selection process may start from a biased state. This observation motivates the use of an external clean seed set to guide the online refinement stage. In this work, ANNE is adopted as the online refinement framework because it already provides a heterogeneous selection mechanism based on feature structure and neighborhood relationships. The main difference is that an offline CLIP-based clean seed set is injected before online refinement so that ANNE can be initialized with more reliable supervision.

### 2.3. Vision–Language Priors for Noisy-Label Cleaning

Recently, vision–language models have provided a new source of external semantic knowledge. CLIP learns transferable visual representations from large-scale image–text pairs and has shown strong zero-shot recognition ability [3]. Since CLIP is pre-trained independently of the target noisy dataset, its predictions are less directly affected by the corrupted labels used during downstream training. This property makes CLIP attractive for noisy-label cleaning, where the dependence on the in-training classifier is one of the main sources of confirmation bias.

CLIPCleaner is one of the first works to use CLIP for noisy-label sample selection [4]. A CLIP-based zero-shot classifier is constructed, and clean samples are selected according to complementary criteria, including prediction consistency and loss modeling based on CLIP-induced probabilities. Since the selection process is performed offline and is decoupled from the downstream classifier, the influence of noisy labels on the sample selection stage can be reduced. This provides a useful external prior for LNL.

However, CLIP-based zero-shot predictions are not always sufficient for the whole noisy-label learning process. Domain gaps may exist between the CLIP pre-training data and the target dataset. Fine-grained categories or visually ambiguous classes may also reduce the reliability of zero-shot predictions. In addition, a conservative CLIP-based selector usually prioritizes precision and may have limited recall, which means that many clean but difficult samples may not be selected at the initialization stage.

These observations indicate that CLIP-based cleaning is more suitable as a reliable initialization mechanism than as a complete replacement for adaptive noisy-label learning. A CLIP-based selector can provide a high-precision subset that is less affected by the in-training classifier, but its conservative nature may limit sample coverage. Online refinement methods, in contrast, can adapt to the target data distribution and recover additional samples, but their early decisions may be affected by unreliable model predictions. CANNE is built on this complementarity. The CLIP-based stage supplies a high-confidence seed set, and the ANNE stage further refines sample partitions using the evolving feature and neighborhood structure of the target model.

## 3. Method

### 3.1. Formulation

Consider a noisy training dataset $D=\{(x_{i},{\tilde{y}}_{i}){\}}_{i=1}^{N}$ with c classes, where $x_{i}\in R^{3\times H\times W}$ denotes an input image and ${\tilde{y}}_{i}\in \{1,\ldots ,C\}$ denotes its observed label, which may be corrupted. Here, H and W indicate the height and width of the image, respectively. The corresponding clean ground-truth label is denoted by $y_{i}\in \{0,1{\}}^{c}$. Due to the presence of annotation noise during the data collection, cases where $y_{i}\neq {\tilde{y}}_{i}$ are common in the dataset D. The goal of LNL is to train a robust DNN under such noisy conditions. Assume that the DNN consists of a feature extractor $f_{\phi }$ and a classifier $g_{\theta }$, where φ and θ are the parameters, respectively. For an input sample $x_{i}$, its feature representation is given by $z_{i}=f_{\phi }\left(x_{i}\right),$ and the prediction on the probability simplex is $p_{i}=g_{\theta }\left(z_{i}\right)$, where $p_{i}\in {\left[0,\;1\right]}^{c}$ The corresponding hard label prediction is denoted as ${\hat{y}}_{i}=\arg\;\underset{k}{\max}\;p_{i}(k)$.

The proposed method, CANNE, is designed to address three common types of label noise: symmetric noise, asymmetric noise, and open-set noise. Symmetric noise is generated by replacing the clean label with a label sampled uniformly from the label space according to a given noise ratio, following the commonly used noisy-label protocol. Asymmetric noise, on the other hand, denotes the case where a clean label is flipped, with probability η, only to visually similar classes (i.e., similar-looking classes). This type of noise is class-dependent, meaning that the transition probabilities depend on the class rather than the input features. Open-set noise is one of the most challenging types, as the true class of a sample may not belong to the predefined label space of the dataset, i.e., the sample may originate from unknown classes outside the training distribution.

**Figure 1:**
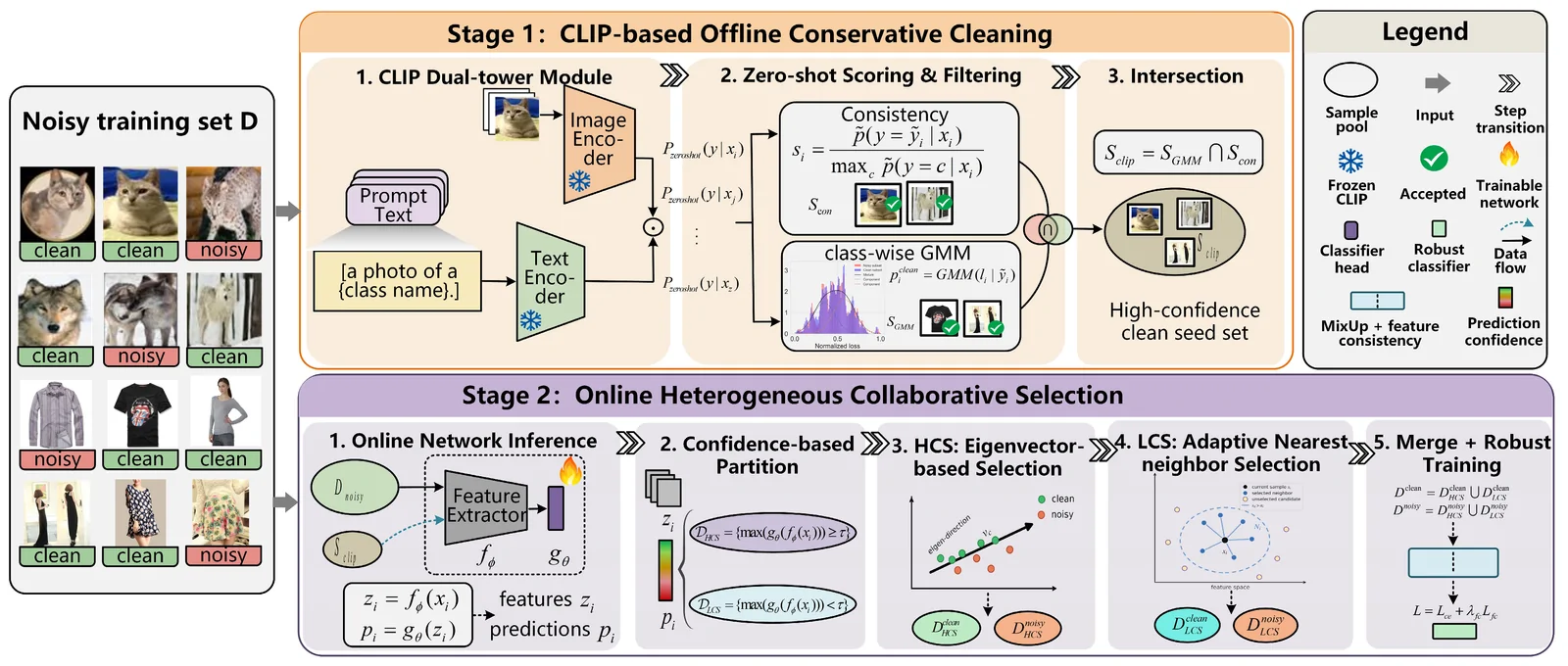
Overview of the proposed CANNE framework. A high-confidence clean seed set is first constructed through CLIP-based conservative offline cleaning and then injected into ANNE-based online refinement for adaptive clean/noisy sample selection and robust training.

The overall framework of CANNE is illustrated in Figure 1. The proposed method consists of two main stages. In the first stage, CLIP is used as an external vision–language prior to perform conservative offline sample cleaning. Specifically, class-wise loss modeling and prediction consistency filtering are jointly used to construct a high-confidence clean seed set D_clip. In the second stage, this clean seed set is injected into the ANNE-based online refinement process. ANNE dynamically partitions the training samples into high-confidence and low-confidence subsets and applies heterogeneous selection strategies to identify clean and noisy samples. By combining the offline CLIP-based clean seeds with online feature-based refinement, CANNE aims to reduce early-stage confirmation bias while preserving the adaptive sample recovery ability of ANNE.

### 3.2. CLIP-Based Offline Sample Cleaning

To reduce the dependence of early sample selection on the in-training model, a CLIP-based offline sample cleaning module, denoted as CSC, is applied before ANNE training. The design follows the conservative sample-selection principle of CLIPCleaner [4], where CLIP is used as an external vision–language model to estimate the compatibility between each image and its observed label. In CANNE, the selected samples are not treated as the final clean training set. Instead, they form a high-confidence seed set that provides reliable anchors for the subsequent ANNE-based online refinement.

Let $D={\left(x_{i},{\tilde{y}}_{i}\right)}_{i=1}^{N}$ denote the noisy training set, where $x_{i}$ is the i-th image and ${\tilde{y}}_{i}\in \{1,...,C\}$ is its observed label. Here, C is the number of classes. CLIP consists of an image encoder $E_{\theta_{v}}$ and a text encoder $E_{\theta_{t}}$. In our implementation, the OpenAI CLIP model with the ViT-B/32 visual backbone is used by default. CLIP is used only for inference, and all its parameters are frozen throughout the whole process. No CLIP fine-tuning, prompt tuning, or knowledge distillation is performed. Images are processed by the standard preprocessing pipeline returned by the OpenAI CLIP implementation, including resizing, center cropping, and CLIP normalization. This keeps the offline cleaning stage independent of the noisy-label training process.

For each class c, a set of descriptive prompts $\{P_{c,j}{\}}_{j=1}^{J}$ is constructed. The prompt template follows the form “a photo of a {class name} {class-specific description}”. The class-specific descriptions are short semantic attributes associated with the class, such as object parts, shapes, or typical visual properties. For example, prompts may include “a photo of an airplane which has wings and a fuselage”, “a photo of an automobile which has four wheels”, “a photo of a ship which is a vessel on water”, and “a photo of a cat which has whiskers and pointed ears”. The prompt set is fixed before training and is not optimized using the noisy labels.

For an image $x_{i}$ and the j-th prompt $P_{c,j}$ of class c, CLIP computes the image–text similarity through the inner product between the encoded image and text features. Following the standard CLIP inference procedure, both image and text features are L2-normalized before computing the similarity. For notational simplicity, $E_{\theta_{v}}(x_{i})$ and $E_{\theta_{t}}(P_{c,j})$ are used below to denote the normalized image and text features, respectively. The multi-prompt class score for class c is defined as:

(1) $$s_{i,c}^{zs}=\sum_{j=1}^{J}exp\left(\frac{E_{\theta_{v}}(x_{i}{)}^{⊤}E_{\theta_{t}}(P_{c,j})}{T}\right),$$

where T is the CLIP temperature and is set to 0.07. The superscript “zs” denotes the zero-shot probability source. The zero-shot conditional probability is then obtained by normalizing the class scores over all C classes:

(2) $$p_{i,c}^{zs}={\tilde{P}}^{zs}(y=c|x_{i})=\frac{\sum_{j=1}^{J}exp\left(E_{\theta_{v}}(x_{i}{)}^{⊤}E_{\theta_{t}}(P_{c,j})/T\right)}{\sum_{k=1}^{C}\sum_{j=1}^{J}exp\left(E_{\theta_{v}}(x_{i}{)}^{⊤}E_{\theta_{t}}(P_{k,j})/T\right)}.$$

Here, $P_{c,j}$ represents the j-th prompt of class c, and J is the number of prompts per class. The probability vector of sample x_i obtained from CLIP zero-shot prediction is denoted as $p_{i}^{zs}=[p_{i,1}^{zs},...,p_{i,C}^{zs}]$.

In addition to the zero-shot probability $p_{i}^{zs}$, CSC also uses an auxiliary probability estimated from frozen CLIP visual features. Specifically, a balanced logistic regression classifier is trained on the frozen CLIP image features using the observed labels, and its output probability vector is denoted as $p_{i}^{lr}$. This induced classifier is used only in the offline cleaning stage and does not update CLIP. The zero-shot probability $p_{i}^{zs}$ and the induced probability $p_{i}^{lr}$ provide two complementary reliability estimates: the former exploits image–text semantic alignment, while the latter adapts frozen CLIP visual representations to the target label space.

The two probability sources are not assumed to be individually reliable enough to define the final seed set. The zero-shot source is independent of the observed training-label distribution but can be affected by prompt wording and image–text domain mismatch. The visual-LR source adapts the frozen visual representation to the target classes but is trained using the observed noisy labels and can therefore inherit label corruption. CSC uses their intersection because a sample supported by both sources is less likely to be selected due to a source-specific error. The contribution of each source is examined through controlled experiments in Section 4.4.3.

Based on the CLIP-derived probability $p_{i}^{r}$, where r∈{zs,lr}, two complementary sample selection criteria are introduced: class-wise loss modeling and prediction consistency filtering. These two criteria evaluate label reliability from different perspectives. The loss-based criterion measures the compatibility between the observed label and the estimated class probability, while the consistency criterion checks whether the observed label agrees with the most confident prediction. The final CSC seed set is obtained by conservatively intersecting the selected subsets so that precision is prioritized at the initialization stage.

(a) Loss-based filtering with class-wise Gaussian mixture modeling

The first criterion evaluates how compatible the observed label is with the CLIP-derived prediction. For each CLIP-derived probability vector $p_{i}^{r}$, where r∈zs,lr, the cross-entropy loss score of sample $x_{i}$ with respect to its observed label ${\tilde{y}}_{i}$ is defined as

(3) $$l_{i}^{r}=-\log p_{i,{\tilde{y}}_{i}}^{r}.$$

Clean samples are expected to have smaller loss values because their observed labels are more consistent with the CLIP-derived prediction. To reduce the influence of class imbalance and inter-class score-scale differences, the loss distribution is modeled separately for each observed class. For class c, let $I_{c}=i|{\tilde{y}}_{i}=c$ denote the index set of samples whose observed label is c. The loss values $l_{i}^{r}|i\in I_{c}$ are assumed to follow a bimodal distribution corresponding to clean and noisy samples and are fitted by a two-component Gaussian mixture model:

(4) $$p(l_{i}^{r}|{\tilde{y}}_{i}=c)=\sum_{m=1}^{2}\pi_{c,m}^{r}N\left(l_{i}^{r};\mu_{c,m}^{r},(\sigma_{c,m}^{r}{)}^{2}\right),$$

where $\pi_{c,m}^{r},\mu_{c,m}^{r}$, and $\sigma_{c,m}^{r}$ denote the mixture weight, mean, and standard deviation of the m-th component for class c, respectively. When the score is written as the cross-entropy loss, the component with the smaller mean is regarded as the clean component. Let $m_{c}^{r}=\arg\underset{m\in \{1,2\}}{\min}\mu_{c,m}^{r}$ denote the clean component for class c. The posterior probability that sample $x_{i}$ belongs to this clean component is computed as

(5) $$q_{i}^{r,gmm}=\frac{\pi_{c,m_{c}^{r}}^{r}N\left(l_{i}^{r};\mu_{c,m_{c}^{r}}^{r},(\sigma_{c,m_{c}^{r}}^{r}{)}^{2}\right)}{\sum_{m=1}^{2}\pi_{c,m}^{r}N\left(l_{i}^{r};\mu_{c,m}^{r},(\sigma_{c,m}^{r}{)}^{2}\right)},$$

where c is the observed class satisfying ${\tilde{y}}_{i}=c$.

A sample is selected by the loss-based criterion if

(6) $$q_{i}^{r,gmm}\geq \tau_{gmm},$$

where $\tau_{gmm}$ is the GMM posterior threshold. Unless otherwise specified, $\tau_{gmm}$ is set to 0.5 in the main experiments.

In implementation, the class-wise scores are normalized before GMM fitting to improve numerical stability. Equivalently, when the normalized log-probability confidence score is used instead of the cross-entropy loss, the clean component corresponds to the component with the larger mean, which is monotonic to the negative cross-entropy loss. This does not change the selection principle. Since the evaluated datasets contain sufficient observed samples for each class, the class-wise GMM fitting is stable in our experiments. In the reported experiments, the conservative intersection produces non-empty seed sets for all classes. Therefore, no additional test-set information or manual class-specific adjustment is used.

(b) Prediction consistency filtering with a fixed threshold

The second criterion evaluates the consistency between the observed label and the most confident CLIP-derived prediction. For each probability source $p_{i}^{r}$, where r∈{zs,lr}, the predicted class of sample $x_{i}$ is defined as

(7) $${\hat{y}}_{i}^{r}=\arg\underset{k\in \{1,\ldots ,C\}}{\max}p_{i,k}^{r}.$$

The consistency score is then computed as

(8) $$a_{i}^{r}=\frac{p_{i,{\tilde{y}}_{i}}^{r}}{\underset{k\in \{1,\ldots ,C\}}{\max}p_{i,k}^{r}}.$$

This score measures the relative agreement between the observed label and the most confident prediction. If the observed label is consistent with the dominant prediction, $a_{i}^{r}$ tends to be close to 1; otherwise, it becomes smaller. A sample is selected as clean by the consistency criterion if

(9) $$a_{i}^{r}\geq \tau_{cons},$$

where $\tau_{cons}$ is a fixed consistency threshold. Unless otherwise specified, $\tau_{cons}$ is set to 0.8 in the main experiments.

Finally, to improve seed precision, CSC conservatively aggregates the selected samples by intersection. Specifically, for both the CLIP zero-shot probability and the CLIP-feature-induced probability, a sample is retained only when it satisfies both the loss-based GMM criterion and the prediction consistency criterion. The resulting high-confidence clean subset is denoted as $D_{clip}$, which is used as the initial clean seed set for the subsequent ANNE-based online refinement. This conservative strategy prioritizes precision over recall, while clean but difficult samples not selected at this stage can still be recovered during the online refinement stage.

### 3.3. Integration with ANNE Framework

After obtaining the initial clean seed set $D_{clip}$ from CSC, we integrate it into the ANNE [5] framework to achieve a fusion of offline and online sample selection. The CLIP-selected subset is not used to replace the original noisy training set. Instead, it provides high-confidence seed anchors for the subsequent online refinement. During the ANNE stage, the entire noisy training set is still processed, and additional reliable samples are progressively identified by jointly exploiting model confidence and feature structure.

Specifically, let $f_{\phi }$ denote the feature extractor and $g_{\theta }$ denote the classifier. For each sample $x_{i}$, the network predicts a softmax probability vector $p_{i}=g_{\theta }(f_{\phi }(x_{i}))$.

Based on the maximum posterior confidence, the noisy training set is partitioned into a high-confidence set D_HCS and a low-confidence set D_LCS as

(10) $$\begin{array}{rr} D_{HCS} & =\left\{(x_{i},{\tilde{y}}_{i})\in D\;|\;\underset{k\in \{1,\ldots ,C\}}{\max}p_{i,k}\geq \tau_{conf}\right\}\\ D_{LCS} & =\left\{(x_{i},{\tilde{y}}_{i})\in D\;|\;\underset{k\in \{1,\ldots ,C\}}{\max}p_{i,k}<\tau_{conf}\right\}, \end{array}$$

Here, $\tau_{conf}$ is estimated by Otsu’s thresholding criterion, which determines the threshold by maximizing the between-group variance of the confidence scores. After the confidence-based partition, two complementary online selection strategies are applied to $D_{HCS}$ and $D_{LCS}$, respectively.

(a) High-Confidence Set (HCS)

Samples in $D_{HCS}$ are expected to contain a relatively high proportion of clean labels. Therefore, a feature decomposition strategy based on class-wise principal directions is adopted. For each class c, let $I_{c}^{HCS}=\left\{i\;|\;(x_{i},{\tilde{y}}_{i})\in D_{HCS},\;{\tilde{y}}_{i}=c\right\}$ denote the index set of high-confidence samples whose observed label is c. The normalized feature of sample $x_{i}$ is defined as $z_{i}=f_{\phi }(x_{i})/||f_{\phi }(x_{i}){\left|\right|}_{2}$. A class-wise scatter matrix is constructed as

(11) $$C_{c}=\sum_{i\in I_{c}^{HCS}}z_{i}z_{i}^{⊤}.$$

Eigenvalue decomposition is then applied:

(12) $$C_{c}=U_{c}\Lambda_{c}U_{c}^{⊤}.$$

Here, $C_{c}$ is a symmetric positive semi-definite scatter matrix, $U_{c}$ contains the orthonormal eigenvectors, and $\Lambda_{c}$ is the diagonal matrix of eigenvalues. The first principal direction $u_{c}$, i.e., the eigenvector in $U_{c}$ corresponding to the largest eigenvalue, represents the dominant feature direction of class c. For each sample $x_{i}$ belonging to class c, an alignment score is computed as

(13) $$s_{i}^{HCS}=\left|z_{i}^{⊤}u_{c}\right|.$$

This score measures the consistency between the sample representation and the class-wise principal direction. Clean samples are expected to lie closer to the dominant class subspace and thus exhibit higher alignment scores, while noisy samples tend to deviate from this structure. Following ANNE [5], a two-component Gaussian mixture model is fitted to the class-wise alignment scores to divide $D_{HCS}$ into a clean subset $D_{HCS}^{clean}$ and a noisy subset $D_{HCS}^{noisy}$. Since larger alignment scores indicate higher reliability, the component with the larger mean is regarded as the clean component.

(b) Low-Confidence Set (LCS)

For samples in $D_{LCS}$, which are more likely to contain noisy labels, an adaptive nearest-neighbor strategy adopted in ANNE [5] is employed to exploit local neighborhood structure. This strategy is related to the neighborhood-based selection idea in SSR [37]. Given two samples $x_{i}$ and $x_{j}$, their cosine similarity is computed using the normalized features $z_{i}$ and $z_{j}$ as

(14) $$s_{ij}=z_{i}^{⊤}z_{j}=\frac{f_{\phi }(x_{i}{)}^{⊤}f_{\phi }(x_{j})}{{\left\|f_{\phi }(x_{i})\right\|}_{2}{\left\|f_{\phi }(x_{j})\right\|}_{2}}.$$

Instead of using a fixed number of neighbors, ANNE [5] introduces an adaptive mechanism. For each sample $x_{i}$, a similarity threshold $\omega_{i}$ is initialized to a high value and iteratively decreased until a minimum number of neighbors $K_{min}$ is reached. Specifically, the neighborhood of $x_{i}$ is defined as

(15) $$N_{i}=\left\{j\;|\;s_{ij}>\omega_{i},\;j\neq i\right\},K_{i}=\left|N_{i}\right|$$

If $K_{i}<K_{min}$, the threshold $\omega_{i}$ is reduced until $K_{i}\geq K_{min}$. This process allows the neighborhood size to adapt to the local feature density. Once the neighborhood $N_{i}$ is determined, label reliability is evaluated via majority voting:

(16) $${\hat{y}}_{i}^{knn}=\arg\underset{c\in \{1,\ldots ,C\}}{\max}\sum_{j\in N_{i}}\;I\left({\tilde{y}}_{j}=c\right),$$

where I(⋅) denotes the indicator function, which equals 1 if the enclosed condition is satisfied and 0 otherwise. The sample $x_{i}$ is assigned to the clean subset $D_{LCS}^{clean}$ if its observed label agrees with the neighborhood majority prediction, i.e., ${\tilde{y}}_{i}={\hat{y}}_{i}^{knn}$; otherwise, it is assigned to the noisy subset $D_{LCS}^{noisy}$.

Overall, the clean and noisy subsets obtained from $D_{HCS}$ and $D_{LCS}$ provide the online selection results of ANNE. Let $D_{clip}$ denote the high-confidence clean seed set obtained by the CSC module in the offline stage. At each training epoch, ANNE first generates the online clean and noisy subsets, denoted as $D_{c}^{ANNE}$ and $D_{n}^{ANNE}$, respectively:

(17) $$\begin{array}{rr} D_{c}^{ANNE} & =D_{HCS}^{clean}\cup D_{LCS}^{clean},\\ D_{n}^{ANNE} & =D_{HCS}^{noisy}\cup D_{LCS}^{noisy}. \end{array}$$

CANNE then injects the offline CLIP-based clean seeds into the online clean subset. The final clean and noisy subsets used for training are defined as

(18) $$\begin{array}{r} D_{c}=D_{c}^{ANNE}\cup D_{clip},\\ D_{n}=D\setminus D_{c}. \end{array}$$

In this way, the samples selected by the CLIP-based conservative cleaning stage are preserved as reliable clean seeds during online refinement. If a seed sample is assigned to the noisy subset by the online selector, the offline CLIP prior takes precedence. Meanwhile, the remaining samples are still adaptively refined by ANNE according to the evolving model predictions and feature structure. This persistent retention rule does not imply that every CSC seed is guaranteed to be correctly labeled. Instead, it represents a precision-oriented design choice. Persistent retention may preserve a small number of erroneous seeds, but it also prevents a much larger number of reliable anchors from being discarded by an uncertain online selector, especially during training under extremely high label noise. Section 4.4.3 directly evaluates this trade-off by comparing persistent retention with a warm-start-only policy using exactly the same seed set.

Finally, MixUp [13] is applied separately to the final clean subset $D_{c}$ and noisy subset $D_{n}$. Given two samples $\left(x_{i},y_{i}\right)$ and $\left(x_{j},y_{j}\right)$ sampled from the same subset, where $y_{i}$ and $y_{j}$ denote their corresponding label vectors, the mixed sample is constructed as

(19) $$\bar{x}=\lambda x_{i}+(1-\lambda )x_{j},\bar{y}=\lambda y_{i}+(1-\lambda )y_{j},$$

where λ∼Beta(α,α). The augmented datasets are denoted as ${\hat{D}}_{clean}$ and ${\hat{D}}_{noisy}$, respectively. Specifically, the overall objective is defined as

(20) $$L=L_{ce}+\lambda_{fc}L_{fc}$$

where

(21) $$L_{ce}=-\frac{1}{\left|{\hat{D}}_{clean}\right|}\sum_{(\bar{x},\bar{y})\in {\hat{D}}_{clean}}{\bar{y}}^{T}\log\left(g_{\theta }(f_{\phi }(\bar{x}))\right),$$

and

(22) $$L_{fc}=\frac{1}{\left|{\hat{D}}_{noisy}\right|}\sum_{\bar{x}\in {\hat{D}}_{noisy}}\left(-\frac{h_{1}(\bar{x}{)}^{T}h_{2}(\bar{x})}{\|h_{1}(\bar{x}){\|}_{2}\|h_{2}(\bar{x}){\|}_{2}}\right).$$

Here, $h_{2}$ denotes the projector output based on the encoder features, and $h_{1}$ denotes the corresponding predictor output. The negative cosine similarity loss encourages feature consistency on the noisy subset. This asymmetric projector-predictor design follows ANNE [5] and helps stabilize training while preventing representation collapse.

Overall, CANNE integrates offline CLIP-based conservative cleaning with online ANNE-based adaptive refinement. CSC constructs a high-precision clean seed set before downstream training, whereas ANNE continuously updates the clean/noisy partition according to the evolving model predictions and feature structure. The CSC seeds are persistently included in the clean subset, while samples outside the seed set remain subject to online selection. This design does not eliminate all seed errors; rather, it balances the risk of retaining a small number of incorrect anchors against the benefit of preserving a substantially larger number of reliable anchors.

## 4. Experiments

### 4.1. Datasets and Experimental Details

To evaluate CANNE under both synthetic and naturally occurring label noise, experiments are conducted on CIFAR-10, CIFAR-100 [47], Animal-10N [48], and Mini-WebVision [2,49]. CIFAR-10 and CIFAR-100 each contain 50,000 training images and 10,000 clean test images. CIFAR-10 contains 10 classes, whereas CIFAR-100 contains 100 finer-grained classes. For closed-set symmetric noise, each training label is independently selected for replacement with probability ρ, after which the replacement label is uniformly sampled from the complete class space. Because the sampled class may coincide with the original class, the effective proportion of changed labels can be slightly lower than the nominal noise ratio. The same generated noise file is reused by all reproduced and controlled variants under each matched experimental setting. Under asymmetric noise, labels are changed according to a predefined class-dependent transition between visually similar categories. The clean test labels are used only for evaluation and are not accessed by CSC or the training algorithm. For open-set noise on CIFAR-10, part of the training set is replaced by images outside the target CIFAR-10 class space and assigned observed labels from the target classes. Closed-set and open-set noise ratios are reported separately in the corresponding experiment. Animal-10N contains 50,000 training images and 5000 test images from 10 animal categories. Its noisy training annotations are naturally collected rather than synthetically generated. No clean training labels are used by CANNE. To broaden the real-world evaluation, we additionally use the commonly adopted 50-class Mini-WebVision protocol. It contains 65,944 web-crawled training images from the first 50 WebVision classes. Evaluation is conducted on both the 2500-image WebVision validation set and the corresponding 2500-image ImageNet validation subset. The two validation sets provide in-domain and cross-domain evaluations, respectively. Since clean ground-truth labels are unavailable for the Mini-WebVision training set, seed precision is not reported for this experiment. The statistics of the datasets are summarized in Table 1.

The experimental settings are summarized in Table 2. The backbone configurations include architectures from the ResNet [50] and VGG [51] families. For CIFAR-10 and CIFAR-100, the model is trained using SGD with a momentum of 0.9 and a weight decay of $5\times 10^{-4}.$ The initial learning rate is set to 0.02. For Animal-10N, the initial learning rate is set to 0.01, and the weight decay is set to $1\times 10^{-3}$. The batch size, warm-up epochs, online refinement epochs, and other hyperparameters follow the settings reported in Table 2. For fair comparison, the same noisy-label settings and evaluation protocols as previous methods are adopted whenever possible. All results are evaluated on the corresponding clean test or validation sets. For CIFAR-10, CIFAR-100, and Animal-10N, “test accuracy” refers to Top-1 classification accuracy. Following the common Mini-WebVision evaluation protocol, both Top-1 and Top-5 accuracies are reported on the WebVision and matched ImageNet validation subsets. For CANNE, we report both the best accuracy achieved during training and the last accuracy obtained at the end of training. When only one value is available for a compared method, we retain the result format reported in the corresponding publication.

The proposed method is compared with representative noisy-label learning methods, including standard cross-entropy training, DivideMix [2], ELR+ [52], FINE [36], LongReMix [26], C2D [53], SSR+ [37], C2MT [38], ANNE [5], and other related methods.

For the CSC module, the OpenAI CLIP model with the ViT-B/32 visual backbone is used by default. The CLIP image and text encoders are kept frozen, and no gradient is propagated to CLIP during the whole training process. For each training sample, two CLIP-derived probability distributions are computed, including the CLIP zero-shot probability and the CLIP-feature-induced probability obtained from a balanced logistic regression classifier trained on frozen CLIP visual features. The class-wise GMM fitting is performed separately under each probability source. Unless otherwise specified, the GMM posterior threshold is set to $\tau_{gmm}=0.5$, and the consistency threshold is set to $\tau_{cons}=0.8.$ These thresholds are fixed before training and are not used as learnable parameters. The resulting clean seed set $D_{clip}$ is computed once before ANNE training and then preserved as reliable clean seeds throughout online refinement. No test-set information is used in CSC.

The influence of $\tau_{gmm}$ and $\tau_{cons}$ is examined separately through a sensitivity analysis on CIFAR-10 with 90% symmetric noise. For the repeated-run analysis, three matched random seeds are used. This follows the common evaluation practice in noisy-label learning, where repeated results are typically reported over a small number of independent runs due to the high cost of full noisy-label training [4,5,26].

### 4.2. Experiments on Synthetic Noisy Datasets

This section evaluates CANNE on synthetic noisy-label benchmarks, including CIFAR-10 and CIFAR-100. Different symmetric noise ratios are considered to examine the robustness of the proposed method under increasing noise severity. Asymmetric noise is also evaluated on CIFAR-10 to test the method under class-dependent label corruption. In addition, open-set noise experiments are conducted to further analyze the robustness of CANNE when noisy samples may not belong to the target label space. These experiments also examine whether the CLIP-selected clean seed set can provide reliable guidance for ANNE-based online refinement under different noisy-label conditions.

#### 4.2.1. Results on CIFAR-10

Following the standard setting in noisy-label learning, CIFAR-10 is evaluated under symmetric noise ratios of 20%, 50%, 80%, and 90% as well as 40% asymmetric noise. The results are reported in Table 3. Overall, CANNE achieves competitive performance across all noisy settings and shows more evident advantages under severe symmetric noise, where online sample selection is more likely to suffer from early-stage confirmation bias.

Under 20% and 50% symmetric noise, CANNE achieves best accuracies of 97.0% and 96.3%, respectively, which are comparable to or slightly better than strong recent baselines. More importantly, under high noise ratios, the benefit of introducing CLIP-based conservative cleaning becomes more evident. CANNE obtains 96.6% and 96.3% best accuracies under 80% and 90% symmetric noise, respectively, outperforming ANNE by 0.8% and 1.0%. This indicates that the high-confidence clean seed set constructed by CLIP provides a useful external semantic prior when the online model is more likely to suffer from early-stage confirmation bias.

For 40% asymmetric noise, CANNE achieves a best accuracy of 95.6%, which is close to the best-performing ANNE result. Since asymmetric noise usually flips labels to visually similar classes, the distinction between clean and noisy samples can be more ambiguous. Nevertheless, the proposed method remains competitive, showing that the CLIP-based initialization does not harm the adaptive refinement ability of ANNE.

To examine whether the improvement under severe noise is caused by a particular random seed, the CIFAR-10 experiment with 90% symmetric noise was repeated using three random seeds. The results are reported in Table 4. ANNE obtains an average best accuracy of 95.11%, whereas CANNE reaches 96.12%, giving an average gain of 1.01 percentage points. The final accuracy is also improved from 94.97% to 95.87% on average. Under the paired setting with the same seeds, the improvement is statistically significant for both best accuracy and final accuracy according to a two-sided paired *t*-test, with $p=4.1\times 10^{-4}$ and $p=3.5\times 10^{-2}$, respectively. These repeated-run results indicate that the gain from CSC is not due to a single favorable initialization.

The quality of the CLIP-selected seed set is also reported in Table 4. Under CIFAR-10 with 90% symmetric noise, CSC selects approximately 8000 samples using the default thresholds, and the seed precision remains around 97.7% across different runs. Since the clean/noisy status is known only for synthetic noise, this diagnostic metric is used solely for analysis and is not involved in training. The high seed precision confirms that CSC behaves as intended: it does not aim to recover all clean samples at the offline stage but instead provides a compact and reliable seed set for stabilizing subsequent ANNE-based online refinement.

#### 4.2.2. Results on CIFAR-100

To further evaluate the proposed method in a more challenging setting, experiments are conducted on CIFAR-100. Compared with CIFAR-10, CIFAR-100 contains more classes and fewer samples per class, making clean-sample identification more difficult, especially under high noise ratios. This setting is also more challenging for CLIP-based seed selection because fine-grained visual categories may lead to more ambiguous semantic predictions. The results are shown in Table 5.

CANNE achieves best accuracies of 81.0%, 79.0%, and 73.6% under 20%, 50%, and 80% symmetric noise, respectively. These results improve over ANNE by 0.6%, 0.9%, and 0.6%, suggesting that the CLIP-based clean seed set can also provide beneficial initialization in a more fine-grained classification setting. The improvement is particularly meaningful because CIFAR-100 contains a larger class space, where online sample selection is more sensitive to early model uncertainty.

However, under 90% symmetric noise, CANNE obtains 60.3% best accuracy and 60.1% final accuracy, which remain below the 66.4% result reported for ANNE. We retain this failure case and conduct additional controlled experiments rather than attributing the degradation to a single factor. Under this setting, the full CSC strategy selects 4085 samples, corresponding to 8.17% of the training set. Among them, 3879 samples retain correct observed labels, and 206 are incorrectly labeled, resulting in 94.96% seed precision and 70.81% recall with respect to all correctly labeled training samples. Although the precision remains high, the class-wise coverage is limited: the average number of seeds per observed class is 40.85, and the least covered class contains only 9 seeds (Table 6). The controlled probability-source and seed-retention experiments in Section 4.4.3 provide a more complete explanation. Using only the zero-shot or visual-LR source introduces substantially more erroneous seeds and reduces the best accuracy to approximately 44%. Conversely, using the high-precision full-intersection seeds only during warm-up achieves only 40.06% best accuracy. Persistent retention of the same seeds improves the best accuracy to 60.30%, indicating that the degradation cannot be explained solely by the permanent retention of erroneous seeds. Persistent retention preserves 206 erroneous anchors, but it also protects 3879 correctly labeled anchors from being discarded by the highly uncertain online selector.

The remaining gap relative to ANNE may therefore be associated with multiple factors, including limited and uneven seed coverage, residual seed errors, the fine-grained semantic ambiguity of CIFAR-100, and the difficulty of online refinement under 90% label noise. The CLIP-backbone analysis further supports this interpretation: using ViT-L/14 in the offline CSC stage substantially improves seed precision, recall, and minimum class-wise coverage. These offline diagnostics suggest that CANNE is more likely to benefit when the external seed set provides reliable and well-distributed anchors; however, the default ViT-B/32 configuration remains limited under the combined difficulty of extreme noise and a large fine-grained class space.

#### 4.2.3. Results Under Open-Set Noise

In addition to closed-set label noise, open-set noise is also evaluated on CIFAR-10. In this setting, part of the noisy samples may come from categories outside the target label space, making the problem more challenging than standard closed-set noise. The results are reported in Table 7.

Compared with DivideMix, SSR+, and ANNE, CANNE achieves competitive performance across different combinations of closed-set and open-set noise. In terms of best accuracy, CANNE obtains 96.7%, 96.3%, 95.6%, and 94.4% under the four evaluated settings, respectively. The corresponding final accuracies are 96.6%, 96.2%, 95.4%, and 94.2%, which are higher than or comparable to those of ANNE. These results suggest that the proposed CLIP-based conservative cleaning can help stabilize the online refinement process when open-set noisy samples exist.

The improvement under open-set noise is consistent with the motivation of CANNE. Since CLIP is pre-trained on large-scale image–text data and is independent of the noisy downstream training labels, it can provide an external semantic prior for identifying reliable clean samples before online training. By injecting these clean seeds into ANNE, the model receives more stable supervision during refinement, which helps reduce the effect of open-set noisy samples.

### 4.3. Experiments on Real-World Noisy Datasets

To evaluate CANNE under naturally occurring label noise, experiments are conducted on Animal-10N and Mini-WebVision. Animal-10N contains noisy annotations for 10 animal classes, whereas Mini-WebVision contains web-crawled images from 50 classes and provides both in-domain and cross-domain validation sets. These two datasets complement the synthetic CIFAR experiments and allow us to examine whether CLIP-based seed construction remains applicable when clean training labels are unavailable. Unlike CIFAR datasets with synthetic label corruption, Animal-10N contains real-world noisy labels collected from online images. Therefore, this dataset provides a more practical benchmark for testing whether the proposed CLIP-based seed initialization can benefit noisy-label learning in realistic scenarios.

#### 4.3.1. Results on Animal-10N

The results on Animal-10N are presented in Table 8. In the table, ANNE [5] denotes the result reported in the original paper, while ANNE* denotes our reproduced result under the current experimental setting. CANNE achieves 87.1% final accuracy and 87.7% best accuracy on Animal-10N. Compared with representative noisy-label learning methods, including DivideMix, C2MT, LongReMix, and HMW+, CANNE obtains competitive performance on this real-world noisy-label benchmark.

Under the matched implementation, CANNE improves the reproduced ANNE best accuracy from 87.2% to 87.7%. This 0.5 percentage-point improvement is modest, and we therefore do not interpret it as a large performance advantage. Instead, the result indicates that the CLIP-based seed initialization remains compatible with ANNE under naturally occurring noise and does not impair its online refinement ability. Together with the Mini-WebVision experiment, this result broadens the real-world evaluation beyond a single noisy dataset.

#### 4.3.2. Results on Mini-Web Vision

To further evaluate CANNE under naturally occurring web-label noise, we conduct an additional experiment using the standard 50-class Mini-WebVision protocol [2,49]. The training set contains web-crawled images with unknown and potentially instance-dependent annotation errors. Unlike the synthetic CIFAR experiments, clean training labels are unavailable; therefore, seed precision is neither used during training nor reported for this experiment.

Following the common Mini-WebVision evaluation protocol, the trained model is evaluated on both the WebVision validation set and the matched ImageNet validation subset. The WebVision validation set provides an in-domain evaluation, whereas the ImageNet validation subset measures cross-domain generalization from noisy web images to a cleaner image distribution. Both Top-1 and Top-5 classification accuracies are reported.

For this experiment, InceptionResNetV2 is used as the downstream classification backbone. Images with variable original resolutions are first resized to 320 pixels and then randomly cropped to 299×299 during training. During evaluation, center cropping to 299×299 is applied. The model is optimized using SGD with an initial learning rate of 0.01, momentum of 0.9, weight decay of $1\times 10^{-4}$, and batch size 32. The CLIP-selected seed set is used for one seed-guided warm-up epoch, followed by 150 online refinement epochs. The learning rate is reduced by a factor of 0.1 after epochs 50 and 100. The random seed is fixed to 3047. According to the default configuration, the CSC thresholds are set to $\theta_{\mathrm{gmm}}=0.9$ and $\theta_{cons}=1.0$.

As shown in Table 9, CANNE achieves a best/last Top-1 accuracy of 81.16%/81.04% and a best/last Top-5 accuracy of 93.52%/93.36% on the WebVision validation set. On the matched ImageNet validation subset, the corresponding Top-1 and Top-5 results are 78.48%/77.84% and 93.96%/92.68%, respectively. The small gap between the best and last results indicates relatively stable performance during the late stage of training. Moreover, the results on the matched ImageNet validation subset suggest that the representation learned from noisy web images retains reasonable cross-domain generalization ability.

### 4.4. Ablation and Sensitivity Analysis

#### 4.4.1. Effect of CSC and Seed Aggregation

The contribution of the CLIP-based conservative cleaning module is first examined by removing CSC from the proposed framework. Without CSC, CANNE reduces to the ANNE-based online refinement baseline. In addition, since CSC relies on two thresholds, $\tau_{gmm}$ and $\tau_{cons}$, for conservative seed selection, the sensitivity of these thresholds is further analyzed around the default setting used in the main experiments.

Specifically, on CIFAR-10 with 90% symmetric noise, the accuracy increases from 95.3% to 96.3%. Under 60% open-set noise, the accuracy improves from 93.7% to 94.4%. On CIFAR-100 with 50% symmetric noise, CSC improves the accuracy from 78.1% to 79.0%. These consistent gains demonstrate that the CLIP-based clean seed set provides a useful external semantic prior for the online refinement stage. By supplying high-confidence clean samples before ANNE-based selection, CSC helps reduce early-stage confirmation bias and improves the reliability of subsequent clean/noisy sample partitioning.

To further understand how CSC constructs the initial clean seed set, we compare several seed aggregation strategies. The default Full intersection strategy keeps only samples that are simultaneously selected by four CLIP-derived criteria: zero-shot GMM, zero-shot consistency, visual-LR GMM, and visual-LR consistency, where visual-LR denotes logistic regression trained on CLIP visual features. The Union variant takes the union of these four criteria. The GMM-only variant uses the intersection of the two GMM-based criteria, while the Consistency-only variant uses the intersection of the two consistency-based criteria. For each variant, we report the number of selected CLIP seeds $D_{clip}$, the seed precision, and the class-wise seed coverage. Seed precision is computed using clean labels only for diagnostic analysis under synthetic noise and is not used during training. Avg. seeds/class and Min. seeds/class are computed over the observed noisy-label classes. Following standard noisy-label learning evaluation, end-to-end test accuracy remains the primary performance metric, whereas the seed statistics are reported only for diagnostic analysis under synthetic noise. Since CSC is designed as a conservative high-precision seed construction module rather than a complete clean-sample detector, we focus on seed precision and class-wise coverage instead of treating recall or F1 as the primary evaluation metrics.

As shown in Table 11, the full intersection strategy produces a compact but high-precision seed set. On CIFAR-10 with 90% symmetric noise, the full intersection selects 8005 seeds with 97.6% precision. Replacing the conservative intersection with a union strategy increases the number of selected seeds to 15,728, but the seed precision drops sharply to 59.1%. A similar trend is observed on CIFAR-100 with 90% symmetric noise, where the union strategy selects 10,671 seeds but achieves only 49.9% seed precision, while the full intersection maintains 94.9% precision.

These results confirm that the conservative intersection is necessary for preventing noisy samples from entering the reliable seed set. The goal of CSC is not to maximize the number of selected samples but to construct a compact and high-precision seed set that can serve as reliable anchors for online refinement.

The single-criterion variants also show lower reliability than the full intersection. For example, on CIFAR-100 with 90% symmetric noise, the GMM-only and consistency-only variants obtain seed precisions of 81% and 91.9%, respectively, both lower than the 94.9% precision achieved by the full CSC strategy. These results support the design of CSC: the GMM-based criterion and the consistency-based criterion provide complementary reliability cues, and their intersection yields a more reliable seed set for ANNE-based online refinement.

Together, Table 10 and Table 11 show that CANNE differs from directly applying CLIP-based filtering or ANNE alone. The offline CLIP-based stage is used to identify a high-precision seed set, while the online ANNE-based stage further refines the remaining samples. The conservative intersection in CSC and the subsequent seed-guided refinement are both important for improving robustness under severe label noise.

These observations also help position CANNE with respect to CLIPCleaner [4]. Both methods use CLIP to estimate label reliability independently of the in-training classifier, but the downstream usage of the selected samples is different. CLIPCleaner combines CLIP-based sample selection with MixFix, a semi-supervised training strategy, whereas CANNE preserves the conservative CLIP-selected subset as high-precision seeds during ANNE-based online refinement. Therefore, CSC is used as a reliable initialization and anchoring mechanism rather than as a complete replacement for adaptive noisy-label learning.

The reported results of CLIPCleaner provide useful context. Since CLIPCleaner uses MixFix as its downstream training strategy, whereas CANNE uses ANNE-based online refinement, these results are included as reported external references rather than identical-protocol reproductions. The original paper reports CLIP zero-shot accuracies of 89.97% on CIFAR-10, 63.72% on CIFAR-100, and 76.12% on Animal-10N, showing that directly using CLIP as a classifier is not sufficient. CLIPCleaner + MixFix reports 94.23% on CIFAR-10 with 90% symmetric noise and 75.23% on CIFAR-100 with 50% symmetric noise, while CANNE achieves 96.3% and 79.0% under the corresponding settings. On CIFAR-100 with 90% symmetric noise, CLIPCleaner reports 63.11%, whereas CANNE obtains 60.3%. Together with the controlled analyses in Table 6 and Table 12, this result suggests that the remaining degradation may be associated with limited and uneven class-wise seed coverage, residual seed errors, fine-grained semantic ambiguity, and the difficulty of online refinement under extreme noise.

#### 4.4.2. Threshold Sensitivity

**Figure 2:**
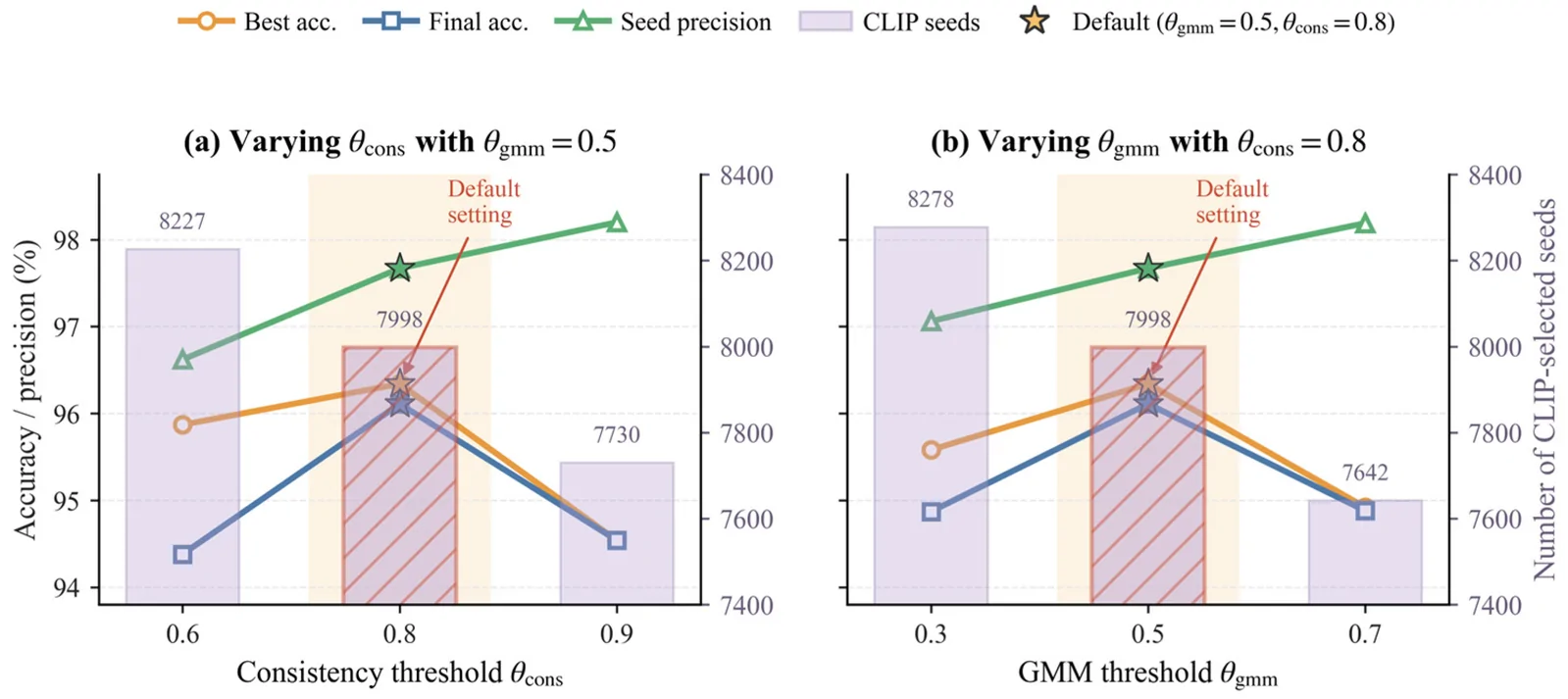
Sensitivity analysis of the two thresholds in the CLIP-based offline cleaning stage on CIFAR-10 with 90% symmetric noise. In (**a**), $\tau_{gmm}$ is fixed to 0.5, and $\tau_{cons}$ is varied; in (**b**), $\tau_{cons}$ is fixed to 0.8, and $\tau_{gmm}$ is varied. The default setting used in the main experiments is $\tau_{gmm}$ = 0.5 and $\tau_{cons}=0.8$, which is highlighted by the shaded region and star markers. The bars denote the number of CLIP-selected clean seeds, while the curves represent the best accuracy, final accuracy, and seed precision. Seed precision measures the proportion of correctly labeled samples among the CLIP-selected clean seeds and is used only for analysis under synthetic noise.

It should be noted that the thresholds are fixed before training and are not tuned using the test set. The following sensitivity analysis is conducted only to examine the robustness of the default setting. The sensitivity results are shown in Figure 2. A clear precision-coverage trade-off can be observed for both thresholds. When the threshold is relaxed, more samples are selected as clean seeds, but the seed precision decreases slightly. For example, reducing $\tau_{cons}$ to 0.6 increases the number of selected seeds to 8227, while the seed precision decreases to 96.62%. In contrast, increasing $\tau_{cons}$ to 0.9 raises the seed precision to 98.20%, but only 7730 seeds are retained. A similar tendency is observed for $\tau_{gmm}$, where a stricter threshold leads to higher precision but lower coverage.

The default setting, $\tau_{gmm}$ = 0.5 and $\tau_{cons}$ = 0.8, provides a balanced operating point. It selects approximately 8000 clean seeds with about 97.6% precision and achieves 96.34% best accuracy and 96.11% final accuracy on CIFAR-10 with 90% symmetric noise. These observations are consistent with the design of CSC: the offline stage is not intended to recover all clean samples but to provide a conservative and reliable seed set that can guide the subsequent online refinement.

We use ViT-B/32 and the class-specific prompt set as the default configuration in the main experiments to avoid test-set-dependent tuning. Because prompt wording and the frozen CLIP encoder may affect offline seed construction, their sensitivity is examined separately in Section 4.4.4 and Section 4.4.5.

#### 4.4.3. Probability-Source and Seed-Retention Analysis

We conduct a controlled diagnostic experiment on CIFAR-100 with 90% symmetric noise because this setting constitutes the main failure case of CANNE. All variants use the same noise file, PreAct ResNet-18 backbone, random seed 3047, batch size 64, 30 seed-guided warm-up epochs, and 300 online refinement epochs.

The zero-shot-only variant constructs its seeds using the loss-based and consistency criteria computed from CLIP zero-shot probabilities. The visual-LR-only variant uses the corresponding two criteria computed from the balanced logistic-regression probabilities. The full variant intersects all four selected subsets. We additionally compare two seed-usage policies. “Persistent” means that the CSC seeds remain in the clean subset throughout online refinement. “Warm-start only” means that the same seeds are used during the 30-epoch warm-up but are no longer forcibly retained during online refinement.

The two individual probability sources produce similar seed-set sizes but contain substantially more incorrect samples. The zero-shot-only seed set contains 567 incorrectly labeled samples, whereas the visual-LR-only set contains 696. Their best accuracies are 44.17% and 43.74%, respectively. Intersecting the two sources reduces the number of incorrect seeds to 206 and increases seed precision to 94.96%. This result confirms that the two sources provide complementary reliability information and that their conservative intersection is important under extreme noise.

The comparison between the two full-intersection variants isolates the effect of persistent retention because both use exactly the same 4085 seeds. Using these seeds only during warm-up yields 40.06% best accuracy. Persistently retaining them increases the best accuracy to 60.30%, an improvement of 20.24 percentage points. Persistent retention therefore involves a trade-off: it also retains 206 erroneous seeds, but it continuously protects 3879 correct anchors. Under this extreme-noise condition, the benefit of preserving the reliable anchors is substantially larger than the adverse effect of the remaining erroneous seeds.

Nevertheless, the resulting 60.30% accuracy remains below ANNE. Thus, this experiment supports the use of the full intersection and persistent retention within CANNE, but it does not eliminate the broader limitation of external seed construction under extremely noisy fine-grained classification.

#### 4.4.4. Prompt Sensitivity

Because text descriptions affect the zero-shot probability source, we evaluate three prompt configurations on CIFAR-10 with 90% symmetric noise: a single class-name prompt, an ensemble of generic CLIP templates, and the default class-specific descriptions (Table 13). Since prompts are used only during the one-time offline CSC stage and are absent from downstream ANNE training, their direct effect is evaluated through zero-shot prediction quality and CSC seed statistics. Clean training labels are used only for this synthetic diagnostic analysis.

The three prompt settings produce similar high-precision seed sets. The generic template ensemble achieves the highest zero-shot accuracy of 88.75%, whereas the class-specific descriptions obtain the highest seed precision of 97.66%. Across all configurations, seed precision remains above 97.1%, approximately 8000 seeds are selected, and no observed class receives an empty seed set. These results indicate that CSC is not critically dependent on one particular prompt wording. The default class-specific prompts favor slightly higher seed precision rather than maximum zero-shot classification accuracy.

#### 4.4.5. Sensitivity to the Frozen CLIP Encoder

We further evaluate RN50, ViT-B/32, and ViT-L/14 using the same class-specific prompt configuration on CIFAR-100 with 90% symmetric noise (Table 14). CLIP remains frozen in all cases. Because the CLIP backbone affects CANNE only through the one-time CSC interface, we report the zero-shot accuracy, visual-LR accuracy, and seed quality rather than retraining the stochastic downstream ANNE model for every frozen encoder.

Seed quality improves consistently across the evaluated frozen CLIP encoders, progressing from RN50 to ViT-B/32 and ViT-L/14. Compared with RN50, ViT-B/32 increases seed precision from 88.12% to 94.96% and recall from 55.93% to 70.81%. ViT-L/14 further improves precision to 97.47%, recall to 82.18%, and the minimum number of seeds per class to 21. These results confirm that CSC depends on the semantic quality of the external vision–language representation. They also support the interpretation that the remaining degradation on CIFAR-100 with 90% noise is partly associated with the limited fine-grained semantic capacity of the default ViT-B/32 encoder. ViT-B/32 is retained as the default model because it provides a more moderate computational cost and is consistent with the main experiments.

### 4.5. Limitations

CANNE has several limitations. First, its seed quality depends on the semantic representation provided by the frozen vision–language model. The backbone experiment shows that weaker CLIP encoders produce lower seed precision and substantially lower class-wise coverage, especially for fine-grained CIFAR-100 categories. Second, the conservative intersection prioritizes precision over recall. This is beneficial in most severe-noise settings but may leave too few reliable anchors for some classes. Third, persistent retention protects reliable seeds but cannot automatically remove every incorrectly selected seed. The controlled experiment shows that its positive effect dominates under the evaluated 90% noise setting, but the remaining erroneous anchors can still restrict final performance. Fourth, the complete CSC stage introduces an external pre-trained model and assumes that meaningful class names or descriptions are available. Its applicability may therefore be reduced for highly specialized domains with weak image–text alignment. Future work may investigate class-aware seed expansion, adaptive retention, automatic prompt construction, and stronger or domain-specific vision–language models.

### 4.6. Computational Cost Analysis

The additional computational cost introduced by CANNE is reported in Table 15. The total runtime includes the complete pipeline, including offline CSC, online ANNE training, sample selection, evaluation, and data-loading overhead. Although CANNE introduces an additional CLIP-based offline cleaning stage, this stage is executed only once before online training.

On CIFAR-10 with 90% symmetric noise, the average total runtime of ANNE is 43,425.23 s, while CANNE takes 45,777.06 s, corresponding to an end-to-end increase of approximately 5.4%. The isolated offline CLIP cleaning stage takes only 63.45 s on average, accounting for about 0.14% of the total CANNE runtime. Therefore, the overall runtime increase is not solely caused by CLIP inference but also includes the complete training pipeline after introducing the seed-guided refinement strategy. Most of the running time is still spent in the ANNE-based online refinement stage. Since $D_{clip}$ is computed once and then reused throughout training, the direct overhead of the offline CSC module remains small compared with the full online refinement process.

## 5. Conclusions

In this paper, a two-stage noisy-label learning framework, termed CANNE, was proposed to combine CLIP-based conservative offline cleaning with ANNE-based adaptive online refinement. CANNE constructs a high-confidence clean seed set using complementary zero-shot and visual-feature-induced probability sources, and it persistently preserves the selected samples as reliable anchors during online refinement.

Experiments on CIFAR-10, CIFAR-100, Animal-10N, and Mini-WebVision, together with evaluation under open-set noise, demonstrate competitive performance across synthetic and naturally occurring noisy-label settings. The repeated-run and runtime results show stable improvements under severe CIFAR-10 noise with moderate end-to-end overhead. Controlled experiments on CIFAR-100 with 90% symmetric noise further show that neither probability source alone provides sufficiently reliable seeds. Their conservative intersection increases seed precision to 94.96%, while persistent retention of the same seed set substantially outperforms using it only during warm-up.

The experiments also reveal the limitations of CANNE. The method remains below ANNE on CIFAR-100 with 90% symmetric noise. The corresponding diagnostics suggest that this gap may be associated with limited and uneven class-wise seed coverage, residual seed errors, fine-grained semantic ambiguity, and the difficulty of online refinement under extreme noise. The prompt analysis indicates that offline seed construction is relatively stable across the evaluated prompt configurations, whereas the CLIP-backbone analysis shows that stronger frozen encoders produce substantially better seed precision and coverage. Future work will investigate class-aware seed expansion, adaptive seed retention, automatic prompt construction, and stronger or domain-specific vision–language priors.

## Figures and Tables

**Table 1:** Overview of the datasets.

Name	Class Number	Training Number	Testing Number	Original Size	Cropped Size
CIFAR-10	10	50 K	10 K	32 × 32	32 × 32
CIFAR-100	100	50 K	10 K	32 × 32	32 × 32
Animal-10N	10	50 K	5 K	64 × 64	64 × 64
Mini-WebVision	50	65,944	5 K	Variable	299 × 299

**Table 2:** Main experimental settings for CIFAR-10, CIFAR-100, and Animal-10N. Here, b denotes the batch size, $E_{w}$ denotes the warm-up epochs, $E_{on}$ denotes the online refinement epochs, and α denotes the MixUp hyperparameter.

Dataset	CIFAR-10	CIFAR-100	Animal-10N
Backbone	PreAct ResNet-18		VGG19-BN/9-layer CNN
Learning rate	0.02	0.02	0.01
Optimizer	SGD	SGD	SGD
Weight decay	5 × 10^−4^	5 × 10^−4^	1 × 10^−3^
Momentum	0.9	0.9	0.9
b	64	64	128
$E_{w}$	10	30	30
$E_{on}$	300	300	200
α	4	4	4

**Table 3:** Comparison of test accuracy (%) on CIFAR-10 under different noisy-label scenarios. The best results are shown in bold.

Methods		CIFAR-10				
		Sym.				Asym.
		20%	50%	80%	90%	40%
Standard CE		86.8	79.4	62.9	42.7	76.1
DivideMix [2]		96.1	94.6	93.2	76.0	93.4
ELR+ [52]		95.8	94.8	93.3	78.7	93.0
FINE [36]		91.0	87.3	69.4	-	89.5
LongReMix [26]		96.3	95.1	93.8	79.9	94.7
C2D [53]		96.4	95.3	94.4	93.6	93.4
SSR+ [37]		96.7	96.1	95.6	95.2	95.5
C2MT [38]	best/last	96.5/96.1	95.0/94.8	93.4/92.8	-	92.9/92.6
ANNE [5]		96.9	96.2	95.8	95.3	**95.7**
Ours	best/last	**97.0/97.0**	**96.3/96.1**	**96.6/96.5**	**96.3/96.1**	**95.6/95.5**

**Table 4:** Repeated-run results on CIFAR-10 with 90% symmetric noise. The reported values are percentages. Seed precision denotes the proportion of correctly labeled samples among the CLIP-selected clean seeds, which is used only for analysis under synthetic noise and is not required during training.

Method	Seed	Best Acc. (%)	Final Acc. (%)	CLIP-Selected Seeds	Seed Precision (%)
ANNE	2024	95.14	95.04	–	–
ANNE	2025	95.30	95.06	–	–
ANNE	3047	94.89	94.82	–	–
ANNE	Mean ± Std.	95.11 ± 0.21	94.97 ± 0.13	–	–
CANNE	2024	96.15	96.12	8002	97.66
CANNE	2025	96.34	96.11	7998	97.67
CANNE	3047	95.86	95.37	7994	97.69
CANNE	Mean ± Std.	96.12 ± 0.24	95.87 ± 0.43	7998 ± 4	97.67 ± 0.02

**Table 5:** Comparison of test accuracy (%) on CIFAR-100 under different symmetric noisy-label settings. The best results are shown in bold. For methods that report two values, the notation “best/last” denotes the best and final test accuracies, respectively.

Methods		CIFAR-100			
		Sym.			
		20%	50%	80%	90%
Standard CE		62.0	46.7	19.9	10.1
DivideMix [2]		77.3	74.6	60.2	31.5
ELR+ [52]		77.6	73.6	60.8	33.4
FINE [36]		70.3	64.2	25.6	-
LongReMix [26]		77.9	75.5	62.3	34.7
C2D [53]		78.7	76.4	67.8	58.7
SSR+ [37]		79.7	77.2	71.9	**66.6**
C2MT [38]	best/last	77.5/76.6	74.2/73.1	57.7/57.5	-
ANNE [5]		80.4	78.1	73.0	66.4
CANNE (ours)	best/last	**81.0/81.0**	**79.0/77.8**	**73.6/73.4**	60.3/60.1

**Table 6:** Diagnostic analysis of the CSC seed set under severe noise settings. Seed precision denotes the proportion of correctly labeled samples among the selected CLIP seeds. This metric is used only for diagnostic analysis under synthetic noise and is not required during training.

Dataset	Noise	$\left|D_{clip}\right|$	Selection Ratio	Seed Precision	Avg./Class	Min. Seeds/Class	Max. Seeds/Class
CIFAR-10	90% Sym.	8005	16.0%	97.6%	800.50	617	904
CIFAR-100	50% Sym.	18,624	37.3%	99.6%	186.24	77	255
CIFAR-100	90% Sym.	4085	8.2%	94.9%	40.85	9	69

**Table 7:** The comparison of test accuracies (%) on CIFAR-10 with open-set noise. Results are reported as best/last, and the best results under each noise setting are shown in bold. Tied best results are also highlighted in bold.

Methods	CIFAR-10				
	Closed Noise	0.15	0	0.3	0
	Open-Set Noise	0.15	0.3	0.3	0.6
EDM [54]		94.5/94.0	92.9/91.9	93.4/92.8	90.6/89.4
DivideMix [2]		91.5/90.9	89.3/88.7	91.8/91.5	89.0/88.7
SSR+ [37]		96.3/96.2	96.1/96.0	95.2/95.2	94.0/93.9
ANNE [5]		96.6/96.4	**96.3/96.2**	95.2/95.0	93.9/93.7
Ours	best/last	**96.7/96.6**	**96.3/96.2**	**95.6/95.4**	**94.4/94.2**

**Table 8:** Comparison of test accuracies (%) on Animal-10N. The notation “best/last” indicates the best and final test accuracies, respectively. When only one value is reported, we follow the result format used in the original paper. The highest reported test accuracy is shown in bold. ANNE denotes the result reported in the original publication, whereas ANNE* denotes our reproduced result under the current experimental setting.

Methods		Test Accuracy (%)
CE		79.4
DivideMix [2]		86.20/85.35
LongReMix [26]		87.22/86.88
C2MT [38]		85.9/85.8
ANNE [5]		**88.2**
ANNE*		87.2
HMW+ [31]		86.5
Ours	best/last	**87.7/87.1**

**Table 9:** Results of CANNE on the 50-class Mini-WebVision benchmark. Top-1 and Top-5 accuracies are reported as best/last (%).

Evaluation Set	Top-1 Best/Last (%)	Top-5 Best/Last (%)
WebVision validation	81.16/81.04	93.52/93.36
ImageNet validation	78.48/77.84	93.96/92.68

**Table 10:** Effect of the CSC module. Best test accuracy (%) is reported. Removing CSC reduces CANNE to the ANNE-based online refinement baseline. The best result in each column is shown in bold.

Rows	CSC	CIFAR-10		CIFAR-100
		90% Sym.	60% Open-Set Noise	50% Sym.
1	✗	95.3	93.7	78.1
2	✓	**96.3**	**94.4**	**79.0**

**Table 11:** Diagnostic comparison of different CSC seed construction strategies. The table reports the size, precision, and class-wise coverage of the selected CLIP seed set.

Dataset	Variant	$\left|D_{clip}\right|$	Seed Precision	Avg. Seeds/Class	Min. Seeds/Class
CIFAR-10 90% Sym.	Full intersection	8005	97.6%	800.50	617
CIFAR-10 90% Sym.	Union	15,728	59.1%	1572.80	1335
CIFAR-10 90% Sym.	GMM only	8282	96.2%	828.20	741
CIFAR-10 90% Sym.	Consistency only	9672	90.4%	967.20	646
CIFAR-100 90% Sym.	Full intersection	4085	94.9%	40.85	9
CIFAR-100 90% Sym.	Union	10,671	49.9%	106.71	61
CIFAR-100 90% Sym.	GMM only	5662	81.0%	56.62	29
CIFAR-100 90% Sym.	Consistency only	4466	91.9%	44.66	11

**Table 12:** Controlled probability-source and seed-retention analysis on CIFAR-100 with 90% symmetric noise. Results for the configuration adopted in our method (full-intersection probability source with persistent seed retention) are shown in bold.

Probability Source	Seed Policy	Seeds	Correct	Wrong	Precision	Recall	Best	Final
Zero-shot only	Persistent	5004	4437	567	88.67	81.00	44.17	43.00
Visual-LR only	Persistent	4996	4300	696	86.07	78.50	43.74	42.21
Full intersection	Warm-start only	4085	3879	206	94.96	70.81	40.06	39.22
Full intersection	Persistent	**4085**	**3879**	**206**	**94.96**	**70.81**	**60.30**	**60.10**

**Table 13:** Offline prompt sensitivity on CIFAR-10 with 90% symmetric noise. Accuracy, precision, recall, and F1 are reported as percentages. The best values for zero-shot accuracy, precision, recall, F1, and minimum seeds per class are shown in bold.

Prompt Setting	Zero-Shot Accuracy	Seeds	Precision	Recall	F1	Min. Seeds/Class
Class name only	88.07	8162	97.18	84.52	90.41	674
Generic templates	**88.75**	8201	97.15	**84.89**	**90.61**	**679**
Class-specific descriptions	86.83	8005	**97.66**	83.31	89.92	621

**Table 14:** Sensitivity of offline CSC seed construction to the frozen CLIP encoder on CIFAR-100 with 90% symmetric noise. The best values for ZS accuracy, Visual-LR accuracy, precision, recall, F1, and minimum seeds per class are shown in bold.

Backbone	ZS Accuracy	Visual-LR Accuracy	Seeds	Precision	Recall	F1	Min. Seeds/Class
RN50	40.88	43.33	3477	88.12	55.93	68.43	8
ViT-B/32	62.63	61.13	4085	94.96	70.81	81.13	9
ViT-L/14	**75.83**	**75.73**	4619	**97.47**	**82.18**	**89.18**	**21**

**Table 15:** Runtime comparison between ANNE and CANNE on CIFAR-10 with 90% symmetric noise. The reported values are averaged over three random seeds and include the complete training pipeline. All measurements are conducted on a single NVIDIA RTX 4090 GPU.

Method	Total Time (s)	Total Time (h)	Online Training Time (s)	Offline CLIP Time (s)	Offline/ Total	Relative Overhead
ANNE	43,425.23	12.06	43,034.03	–	–	–
CANNE	45,777.06	12.72	45,561.55	63.45	0.14%	+5.4%

## Data Availability

CIFAR-10 and CIFAR-100 are publicly available at https://www.cs.toronto.edu/~kriz/cifar.html (accessed on 19 July 2026). Animal-10N is available at https://dm.kaist.ac.kr/datasets/animal-10n/ (accessed on 19 July 2026). WebVision is available at https://data.vision.ee.ethz.ch/cvl/webvision/dataset2017.html (accessed on 19 July 2026). The matched ImageNet validation subset follows the standard Mini-WebVision evaluation protocol.
